# Reutilizing Flavonoids from Agricultural By-Products for In Situ Generation and Immobilization of AgNPs on Silk Towards Coloration, Antimicrobial and Anti-UV Functions

**DOI:** 10.3390/ma18235409

**Published:** 2025-11-30

**Authors:** Wei Chen, Yijie Yue, Xiaoqi Zhou, Jingyu Sun, Leyang Chen, Xiaoyan Hu, Yuyang Zhou

**Affiliations:** 1 School of Art, Soochow University, Suzhou 215123, China; chenweisdyz@163.com (W.C.); yueyijie@suda.edu.cn (Y.Y.);; 2 College of Textile and Clothing Engineering, Soochow University, Suzhou 215123, China

**Keywords:** flavonoids, AgNPs, in situ synthesis, silk, coloration, antimicrobial

## Abstract

The utilization of agro-byproducts for textile dyeing and finishing is strongly suggested to meet sustainability and cost-efficiency objectives. Despite recently proliferating studies, three major issues hinder the industrialization of such a technique: identifying reasonable bio-resources, ensuring compatibility between agro-byproducts and textile substrates, and achieving satisfactory color depth, functionality, and durability. This research introduces an approach that forms and fixes silver nanoparticles (AgNPs) on silk using three representative flavonoids (FLs)—Quercetin (QUE), Baicalin (BAI), and Rutin (RUT)—through a single-step in situ bio-reduction. Results demonstrate that FLs-synthesized AgNPs@silk generates attractive spectra of hues, varying from pale cream-brown to deep golden-brown. Using an equivalent quantity of FLs, the color intensity of silk descends in QUE-Ag@silk > BAI-Ag@silk > RUR-Ag@silk, due to the decreasing reactivity and binding affinity of FLs to silk. SEM reveals uniformly distributed spherical AgNPs in dimensions between 20 and 40 nm on silk and the dimension inversely correlates with FLs concentration while being directly proportional to silver nitrate. The modified silk exhibits remarkable antimicrobial performance (>98% pathogen elimination) and exceptional wash resistance (>90% reduction both of *E. coli* and *S. aureus* after ten cycles of washing). Additionally, the FLs-synthesized AgNPs provide silk with superior UV shielding capability. This study stems from environmental awareness and sustainable production of AgNPs by FLs, ready for developing hygienic and therapeutic textile materials.

## 1. Introduction

The textile dyeing and finishing sector encounters mounting difficulties as synthetic chemical usage raises environmental hazards and health concerns. Growing emphasis now lies on creating eco-friendly alternatives for textile processing. Utilizing agricultural waste materials for fabric coloration and enhancement aligns with sustainability objectives and resource conservation efforts. Contemporary investigations into repurposing natural byproduct derivatives have revitalized prospects for replacing toxic synthetic colorants and finishing agents [1,2]. Beyond providing esthetic hues, plant-based extracts confer valuable textile properties including microbial resistance, oxidation prevention, odor control, and ultraviolet shielding capabilities [3]. Despite increasing scholarly attention toward novel botanical extracts and application methodologies, three critical obstacles hinder industrial adoption: identifying sustainable bio-extract origins, ensuring material compatibility, and achieving satisfactory colorfastness, performance consistency, and product longevity.

Flavonoids (FLs), representing the predominant category within natural plant extracts, are biologically active compounds produced by vegetation as protective agents against pathogenic microorganisms. Altıok et al. [4] demonstrates the efficacy of silk fibroin as an innovative adsorption medium for isolating polyphenolic compounds, particularly various FLs, from olive leaf extracts, revealing specific molecular interactions between silk proteins and flavonoid structures. This finding suggests potential applications of FLs in developing antimicrobial silk materials. *Scutellaria baicalensis* Georgi has been a fundamental medicinal herb in traditional Chinese pharmacology for millennia [5]. Its primary bioactive component, baicalin (BAI), is predominantly concentrated in the plant’s root system. A distinctive structural characteristic of BAI involves the diorthohydroxyl functional group located on its A-ring. Quercetin (QUE) and rutin (RUT) represent additional significant members of the flavonoid family, widely distributed in botanical sources such as grape skins, citrus peels, onion bulbs, and various plant tissues [6,7]. Structurally, RUT consists of a glycosylated derivative where the QUE aglycone is covalently linked at the C-3 position (ring C) to rutinose, a disaccharide composed of rhamnose and glucose subunits. Despite these compounds’ biological significance, existing studies indicate limitations when directly applying BAI, QUE, and RUT to silk substrates, particularly concerning color intensity, antimicrobial performance, and material stability over time.

The integration of nano-enhanced materials into consumer products for upcoming commercial applications is gaining significant traction. Silver nanoparticles (AgNPs) stand out among commercialized nanomaterials, capturing interest across diverse sectors such as food processing, textile manufacturing, construction, medical fields, cosmetic industries, and pharmaceutical applications, with their global market experiencing exponential growth [8,9]. These nanoparticles demonstrate exceptional versatility owing to their variable morphology, dimensional range, enhanced stability, expansive surface characteristics, along with distinctive optical, catalytic, biophysical, and antimicrobial attributes. Recent scientific attention has concentrated on developing pigmented and multifunctional textiles utilizing metallic nanoparticles, particularly AgNPs. The chromatic effects observed in textiles treated with AgNPs stem from the localized surface plasmon resonance (LSPR) phenomenon inherent to these nanomaterials [10]. Textile substrates modified with AgNPs showcase multifaceted utility across various textile applications. Traditionally, AgNPs integration into textiles follows a two-stage ex situ approach: initial nanoparticle synthesis followed by subsequent textile impregnation through various techniques. However, this ex situ functionalization presents multiple limitations, including the necessity for protective agents to prevent nanoparticle aggregation during solution preparation, mandatory purification steps prior to application, inconsistent nanoparticle dispersion across fabric surfaces, and durability concerns linked to potential silver leaching during washing or mechanical stress. Conversely, the in situ methodology, involving direct conversion of pre-adsorbed silver ions into AgNPs on textile substrates, offers distinct advantages through simplified processing, resource efficiency, and enhanced nanoparticle adhesion [11,12]. To date, a great number of works in the literature utilize one single type of bio-extract or molecule for AgNPs synthesis and application on textiles [13,14], but there are limited comparative studies examining in situ AgNPs generation on silk fabrics regarding chromatic performance and microbial resistance. Despite increasing research activity over the past decade, three primary challenges persist: sourcing appropriate biological extracts, ensuring byproduct–textile compatibility, and achieving optimal color intensity with sustained functionality. This investigation presents a novel single-step biological reduction technique employing three FLs—BAI, QUE, and RUT—for concurrent AgNPs formation and immobilization on silk substrates. The developed methodology addresses several existing limitations: enhancing the typically faint coloration and subpar antimicrobial performance of flavonoid-treated silk, removing hazardous reducing agents from nanoparticle synthesis, and resolving difficulties in obtaining homogeneous AgNPs distribution across silk fibers. For AgNPs-functionalized silk (AgNPs@silk), comprehensive characterization includes scanning electron microscopy (SEM), energy-dispersive X-ray spectroscopy (EDS), X-ray diffraction (XRD), and Fourier-transform infrared spectroscopy (FTIR). A Response Surface Methodology (RSM) framework incorporating central composite design (CCD) evaluates the synergistic effects of processing parameters on FL-mediated AgNPs deposition. Furthermore, extensive evaluation and comparative analysis of AgNPs@silk encompass chromatic characteristics, wash durability, antimicrobial performance, and ultraviolet protection capabilities.

## 2. Materials and Methods

### 2.1. Materials

The 12103-grade crepe de chine silk material was sourced from Wujiang Zhiyuan Textile Corporation located in Suzhou, China. BAI, QUE, and RUT compounds were acquired from Xi’an Qing Yue Biotech Company in Xi’an, China. Analytical-grade chemical reagents were employed throughout the experiments. For assessing wash resistance, a specialized silk cleaning formulation was utilized. Ultrapure water served as the aqueous medium for all experimental procedures.

### 2.2. Fabrication of AgNPs@silk

The synthesis of AgNPs@silk was performed through an in situ approach with the following procedure. A series of silver nitrate solutions were created by dissolving accurately measured quantities of AgNO_3_ in ultrapure water within conical flasks, which were then positioned in a XW-ZDR low-vibration oscillating dyeing apparatus (manufactured by Jingjiang Xinwang Dyeing and Finishing Machinery Factory, Jingjiang, China). The system was maintained at 30 °C initially. Silk textile samples weighing 1 g were submerged in the prepared silver nitrate solutions. The liquid temperature was gradually increased to 90 °C at a controlled heating rate of 5 °C per minute. Following this, a 2 mL quantity of FLs solution (50% ethanol) at predetermined concentrations ranging from 1 to 4 mM/L was introduced via dropwise addition. The flask openings were sealed with polyethylene film while maintaining constant stirring at 90 °C for a duration of 30 min. Upon completion, the textile specimens underwent multiple rinses with deionized water to eliminate any unbound substances. The final treated silk fabrics were then air-dried under ambient conditions.

### 2.3. CCD of Experiment

The experimental data analysis was conducted using Minitab 19 statistical software (trial edition, State College, PA, USA). Table 1 and Table 2 present the coded variable parameters and factorial arrangement matrix correspondingly. Analysis of variance (ANOVA) was employed to examine both individual factor influences and their interactive effects.

### 2.4. Characterization of AgNPs@silk

The structural characteristics of silk fabric were examined through Hitachi TM3030 benchtop and Hitachi-S4800 field-emission scanning electron microscopes (Hitachi High Technologies America Inc., Clarksburg, MD, USA). The TM3030 system incorporated energy dispersive spectroscopy (EDS) for conducting qualitative assessments of silver particles on the textile surface. Regarding XRD and FTIR analyses, AgNPs@silk specimens were sectioned into fragments before pulverization to create powdered test materials. X-ray diffraction (XRD) patterns of the fabric powders were recorded on an X’Pert-Pro MPD diffractometer (PANalytical B.V., Almelo, The Netherlands) employing CuKα radiation with a 0.15418 nm wavelength. Fourier-transform infrared (FTIR) spectroscopy was performed on silk powder samples using a Nicolet 5700 FT-IR instrument (Thermo Fisher Scientific Inc., Waltham, MA, USA). Colorimetric evaluation of AgNPs@silk included measurement of L (lightness), a (red-green axis), b (yellow-blue axis), C (chromatic intensity), and h (hue angle) parameters, along with apparent color strength (*K/S* values). These measurements were conducted with a HunterLab UltraScan PRO spectrophotometer (HunterLab, Reston, VA, USA) under D65 illumination with a 10° observation angle. For optimal measurement conditions, samples were doubled over to achieve four-layer thickness. Fabric specimens underwent conditioning in controlled environmental settings (maintained at 65 ± 5% humidity and 21 ± 1 °C) for a full day before testing. Mechanical properties were evaluated with an Instron 3365 testing apparatus (manufactured by Illinois ToolWorks Inc., London, UK) following the ISO 13934-1:2013 protocol. Six individual test pieces from each material batch underwent evaluation, with the final data reflecting mean values derived from stress–strain curves. The Ag content was determined by the inductively coupled plasma optical emission spectrometer (ICPOES) ICAP 6300 DUO (Thermo Fisher Scientific Inc., Waltham, MA, USA) with argon plasma at the wavelength of 328.028 nm.

### 2.5. Functionalities

Antioxidant activity: The fabric samples’ antioxidant capacity was evaluated through spectrophotometric measurement of ABTS radical neutralization activity, following an established protocol. The ABTS radical cation was generated by combining ABTS (7 mM) stock solution with potassium persulfate (2.45 mM), then allowing the mixture to stand in darkness at ambient temperature for 12–16 h. This radical maintained stability for over 48 h when kept in dark conditions at room temperature. Prior to testing, the ABTS solution was adjusted with phosphate buffer (0.1 M, pH 7.4) to achieve an optical density reading between 0.675 and 0.725 at 734 nm wavelengths. Subsequently, 10 mg specimens were introduced into 10 mL aliquots of the prepared ABTS solution. Following a 30 min incubation period, the percentage reduction in ABTS absorbance at 734 nm was quantified using Equation (1),

(1) $$\mathrm{Antioxidant}\;\mathrm{activity}\;(\%)=\frac{A_{\mathrm{ctrl}}-A_{\mathrm{spl}}}{A_{\mathrm{ctrl}}}\times 100$$

where *A*_ctrl_ is the initial absorbance of the ABTS and *A*_spl_ is absorbance of the remaining ABTS in the presence of fiber sample.

Antibacterial activity: The antimicrobial efficacy of baicalin-treated silk fabrics was assessed following the GB/T 20944.3-2008 standard protocol. *E. coli* and *S. aureus* were served as the test microorganisms for this investigation. Fabric samples weighing 0.75 g were submerged in conical flasks containing bacterial suspensions, which were then agitated in an orbital shaker under controlled temperature conditions. Following incubation, the bacterial suspensions underwent 1000-fold dilution before being transferred to agar plates for cultivation at 37 °C. After an appropriate incubation period, visible bacterial colonies were enumerated, with antimicrobial performance calculated using Equation (2),

(2) $$\mathrm{Antibacterial}\;\mathrm{activity}\;(\%)=\frac{N_{\mathrm{ctrl}}-N_{\mathrm{spl}}}{N_{\mathrm{ctrl}}}\times 100$$

where *N*_ctrl_ and *N*_spl_ are the quantities of the visually bacterial colonies of standard cotton fabric and tested silk fabric, respectively.

UV protection: The UV and UPF transmission properties of the specimens were evaluated using a Labsphere UV-1000F analyzer (Labsphere Inc., North Sutton, NH, USA). Measurements were conducted at four distinct locations per sample, with the final results representing the mean values obtained.

### 2.6. Durability

The cleaning procedure for processed silk involved submerging the textile materials in a detergent solution comprising 4 g per liter of standard cleaning agent, maintaining a bath ratio of 1:50. Subsequently, the specimens underwent agitation followed by a 10 min dwell period at 40 ± 2 °C within a WashTec-P colorfastness apparatus (manufactured by Roaches International, Leek, UK). Following this, the fabrics were softly pressed and thoroughly flushed with municipal water supply. This sequence was replicated for ten instances to achieve ten complete cleaning cycles.

## 3. Results

### 3.1. CCD Experiment

The AgNPs@silk composite was fabricated through FLs-mediated reduction of silver ions directly on the textile substrate. Initially, silver ions permeated the silk material immersed in AgNO_3_ solution, facilitating their attachment to the silk framework. Upon introduction of FLs solution, the adsorbed Ag^+^ ions triggered immediate nanoparticle formation, causing the fabric’s hue to transition from pale white to a distinctive golden-brown shade. This chromatic shift originates from the surface plasmon resonance characteristics of the produced silver nanoparticles. Research indicates a direct correlation between coloration intensity and AgNPs deposition density on silk surfaces [15,16]. Consequently, the *K/S* measurement, representing color saturation, serves as the primary metric for evaluating and refining the synthesis protocol. Response surface methodology (RSM) represents a statistical approach employed for enhancing and refining processes, facilitating the assessment of how different operational factors influence resultant outcomes. This technique proves particularly valuable when identifying ideal parameter configurations to achieve optimal process performance. Within the realm of natural dye extraction, RSM has demonstrated widespread application for parameter refinement across diverse source materials. Contemporary research further underscores the advantages of implementing RSM techniques for variable optimization in textile coloration processes, though existing literature predominantly examines applications involving cotton, wool, and synthetic fabric substrates.

An extensive investigation employing CCD methodology was conducted to analyze the complex influences affecting the chromatic intensity of FLs-mediated AgNPs@silk composites, providing deeper insights than conventional single-variable approaches. A pivotal mathematical model was developed to characterize the correlation between operational parameters and textile coloration properties. Through advanced response surface analysis techniques, researchers performed thorough evaluations of the experimental dataset. As outlined in Table 1, the investigated factors were A. FLs concentration ranging from 1.32 to 4.68% owf; B. AgNO_3_ concentration spanning from 0.53 to 1.87; and C. temperatures between 53.18 °C and 86.82 °C. Consequently, three equations respective for *QUE-Ag@Silk*, *BAI-Ag@Silk and RUT-Ag@Silk* are established as follows,

Regression Equation for *QUE-Ag@Silk:*

*K*/*S* = −25.35 + 1.484 × Conc-QUE + 14.53 × Conc-Ag + 0.486 × Temp − 0.0600 × Conc-QUE × Conc-QUE − 4.441 × Conc-Ag × Conc-Ag − 0.0029 × Temp × Temp + 0.495 × Conc-QUE × Conc-Ag − 0.0002 × Conc-QUE × Temp − 0.0290 × Conc-Ag × Temp

Regression Equation for *BAI-Ag@Silk:*

*K/S* = −11.24 + 1.849 × Conc-BAI + 11.54 × Conc-Ag + 0.1250 × Temp − 0.1498 × Conc-BAI × Conc-BAI − 4.247 × Conc-Ag × Conc-Ag − 0.00063 × Temp × Temp + 0.458 × Conc-BAI × Conc-Ag − 0.00598 × Conc-BAI × Temp − 0.0044 × Conc-Ag × Temp

Regression Equation for *RUT-Ag@Silk:*

*K/S* = −4.62 + 0.756 × Conc-RUT + 5.147 × Conc-Ag + 0.0461 × Temp − 0.0663 × Conc-RUT × Conc-RUT − 1.8012 × Conc-Ag × Conc-Ag − 0.000216 × Temp × Temp + 0.1508 × Conc-RUT × Conc-Ag − 0.00158 × Conc-RUT × Temp − 0.00461 × Conc-Ag × Temp

Table 3, Table 4 and Table 5 display the experimental matrix alongside computed and measured *K/S* ratios across varying concentrations of FLs, AgNO_3_, and temperature conditions. The statistical analysis reveals F-values of 90.31, 142.99, and 185.94 for *QUE-Ag@Silk*, *BAI-Ag@Silk*, and *RUT-Ag@Silk, respectively,* confirming robust model fitting. Each linear parameter (A, B, C) demonstrates statistical significance with *p*-values under 0.001, emphasizing their substantial influence on silver nanoparticle formation within silk substrates. Notably, the combined effect of FLs concentration and silver ion concentration shows particularly strong interactions for *BAI-Ag@Silk* and *RUT-Ag@Silk* (*p* < 0.001). While *QUE-Ag@Silk* exhibits a less pronounced interaction (*p* > 0.05), it remains substantially lower than alternative parameter combinations. These findings suggest a direct relationship between flavonoid compounds and silver ion reduction, with BAI and RUT demonstrating superior catalytic activity—likely attributable to their enhanced solubility promoting more efficient nanoparticle synthesis. All three models show non-significant lack-of-fit (*p* > 0.05), validating their statistical reliability. Predictive accuracy is further evidenced by *R*^2^ values of 98.78%, 99.23%, and 99.41% for *QUE-Ag@Silk, BAI-Ag@Silk*, and *RUT-Ag@Silk*, respectively, closely matching their adjusted *R*^2^ counterparts. This strong correlation between predicted and experimental data confirms the models’ exceptional predictive capability for future applications.

**Figure 1:**
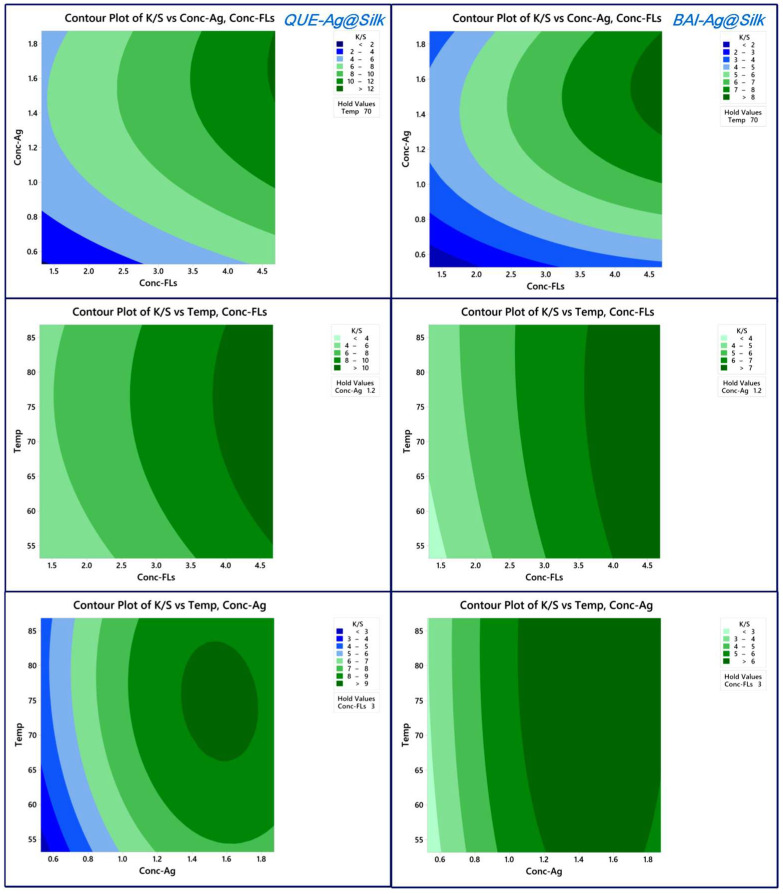
Contour plots for the *K/S* values of QUE-Ag@Silk and BAI-Ag@Silk as functions of conc. of FLs, conc. of Ag^+^ and Temp.

**Figure 2:**
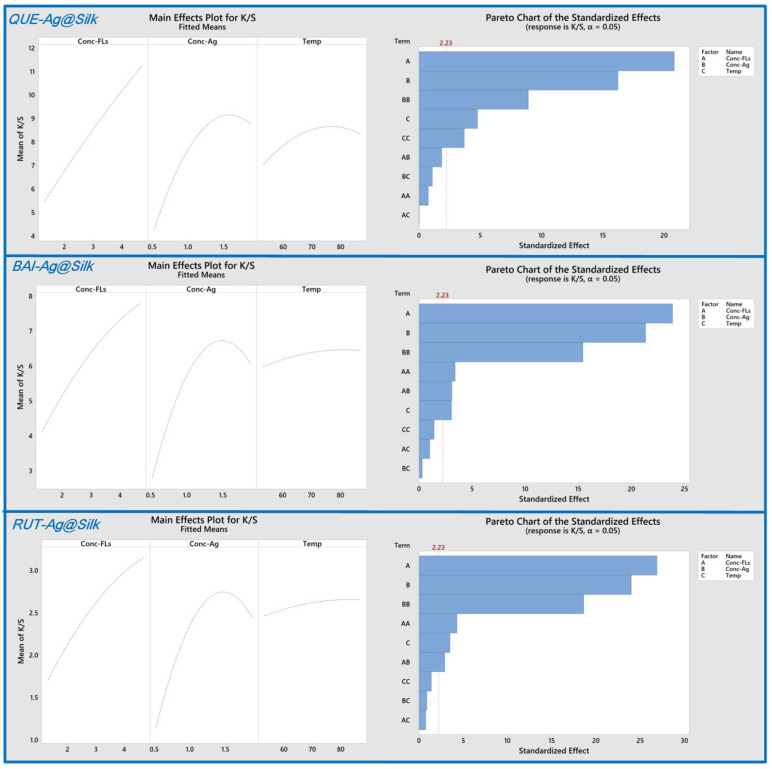
Main effects plot and Pareto Chart for the *K/S* values of QUE-Ag@Silk, BAI-Ag@Silk and RUT-Ag@Silk as functions of conc. of FLs, conc. of Ag^+^ and Temp.

**Figure 3:**
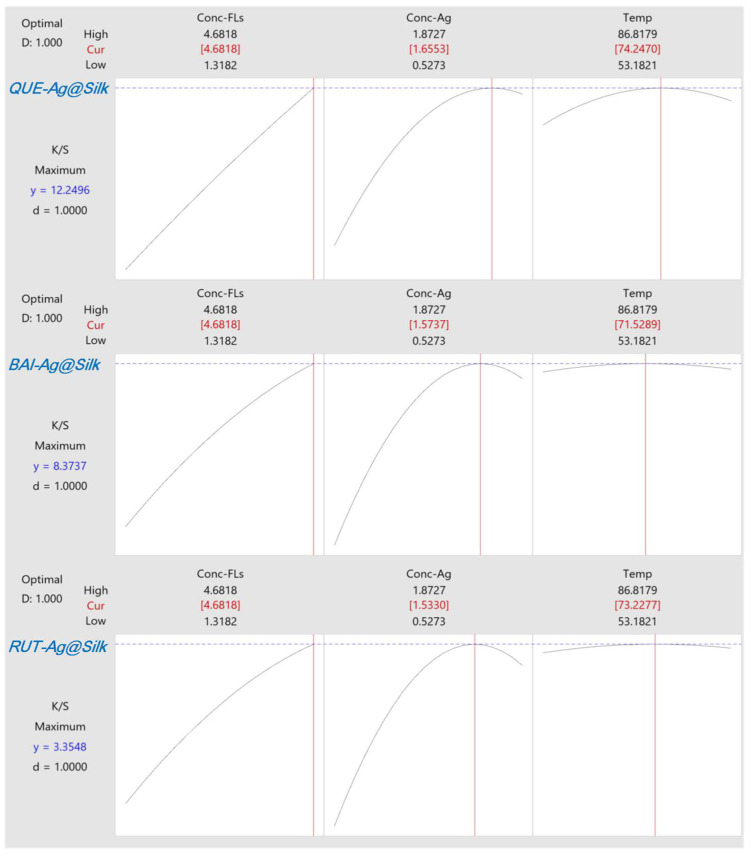
Optimization of *K/S* values against conc. of FLs, conc. of Ag^+^ and Temp.

The influence of parameters was analyzed through 2D contour plots representing experimental *K/S* values derived from the quadratic model. Figure 1 and Figure S1 reveals that the dark green zone, indicating elevated *K/S* measurements, appears in the upper right section of the plot. This demonstrates that optimal *K/S* results occur with increased levels of both FLs and AgNO_3_. However, an oversupply of AgNO_3_ hinders the formation of AgNPs on silk fabric. Elevated temperatures similarly enhance AgNP synthesis by accelerating the reaction kinetics, though exceeding 80 °C diminishes *K/S* values as Ag^+^ converts to Ag_2_O instead of forming nanoparticles. Comparative analysis of the three FLs shows distinct behaviors. BAI and RUT exhibit comparable AgNP formation patterns on silk according to the contour plots, differing from QUE’s performance. This discrepancy arises because BAI and RUT possess superior water solubility, facilitating their adsorption onto silk and subsequent reduction of Ag^+^ to AgNPs. Essentially, the localized production of AgNPs on silk relies not just on the reducing capacity of FLs but also on their accessibility to the silk surface. The primary effects plot (Figure 2) highlights the relative importance of each variable, with FLs and AgNO_3_ concentrations exhibiting steeper slopes than temperature, indicating their greater influence. Pareto analysis (Figure 2) further confirms this trend, ranking the parameters as FLs concentration > AgNO_3_ concentration > temperature. Software-generated optimization results for *QUE-Ag@Silk, BAI-Ag@Silk,* and *RUT-Ag@Silk* are presented in Figure 3. While higher FLs concentrations generally improve outcomes, AgNO_3_ levels and temperature must be carefully regulated within 1.5–1.6 mM and 72–74 °C, respectively, for optimal performance.

### 3.2. FTIR and XRD

**Figure 4:**
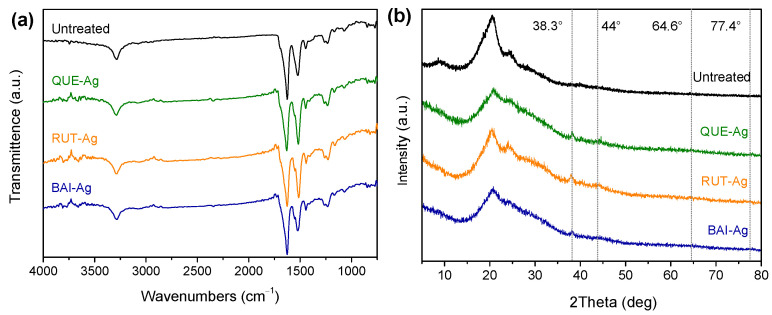
(**a**) FTIR and (**b**) XRD of QUE-Ag@Silk, BAI-Ag@Silk and RUT-Ag@Silk.

Figure 4a presents the FTIR spectral comparison between untreated silk and AgNPs@silk. Distinct vibrational regions are observable in the infrared spectra: 2800–3650 cm^−^^1^ (hydroxyl, amino, and alkyl stretching vibrations), 1200–1800 cm^−1^ (peptide bond vibrations and methylene deformation), 700–1200 cm^−1^ (backbone vibrations), and 400–1300 cm^−1^ (structural deformation modes). The diagnostic amide I (1700–1600 cm^−1^), amide II (1540–1520 cm^−1^), and amide III bands originate from peptide bond vibrations, with the carbonyl stretching being particularly prominent in the amide I region. Both materials exhibited identical spectral features, demonstrating that silver nanoparticle incorporation preserves the molecular architecture of silk despite visible color alterations. X-ray diffraction analysis (Figure 4b) verified the crystalline formation of silver nanoparticles through FL-mediated synthesis, displaying characteristic reflections at 38.3° (111), 44° (200), 64.6° (220), and 77.4° (311) corresponding to the face-centered cubic lattice of metallic silver [17]. These diffraction patterns confirm the crystalline quality of the synthesized nanoparticles and show excellent agreement with reference data (JCPDS 04-0783). The XRD results provide conclusive evidence of successful nanoparticle formation while maintaining the structural integrity of the silk substrate.

### 3.3. Morphological and Tensile Properties

**Figure 5:**
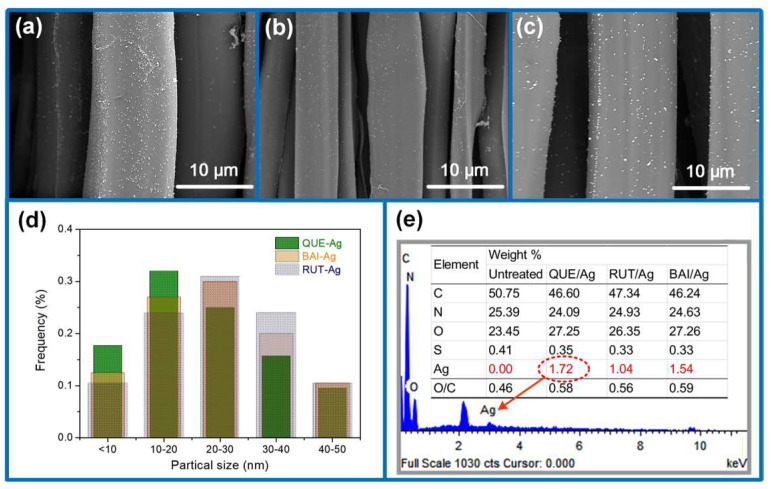
SEM images of (**a**) QUE-Ag@Silk, (**b**) BAI-Ag@Silk, (**c**) RUT-Ag@Silk (**d**,**e**) EDS analysis.

**Figure 6:**
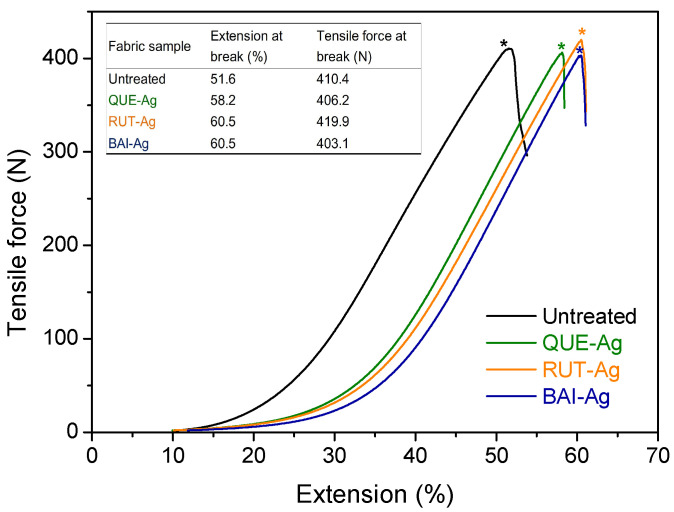
Tensile property of untreated silk, QUE-Ag@Silk, BAI-Ag@Silk and RUT-Ag@Silk. (Note: * represents the peak of the curve).

Scanning electron microscopy at high resolution revealed numerous nanoparticles adhering to the silk fiber’s exterior (Figure 5a–c). Variations in AgNPs dimensions, population density, and clustering patterns correlated with both silver salt concentration and flavonoid levels. The statistical analysis of AgNPs particle size is displayed in Figure 5e according to the SEM image. The average particles of AgNPs are in a descending order: RUT-Ag > BAI-Ag > QUE-Ag. The morphologies of AgNPs are also observed by TEM in Figure S2. Elemental mapping through EDS confirmed elevated silver content on silk substrates following nanoparticle synthesis (Figure 5e). Oxygen-to-carbon ratios exceeded baseline silk values in flavonoid-treated samples, with subsequent increases in O/C ratios for flavonoid-silver combinations suggesting dual surface deposition. Mechanical testing involved recording peak stress values from stress–strain curves at fabric failure points. Reference measurements showed untreated silk exhibiting 410 N mean breaking force and 52% elongation capacity (Figure 6). Silver nanoparticle integration demonstrated minimal influence on tensile properties, while enhanced elongation characteristics originated from textile contraction effects. Such phenomenon is reconfirmed by the area shrinkage of the fabric after wet process (Figure S3).

### 3.4. Color Features

**Figure 7:**
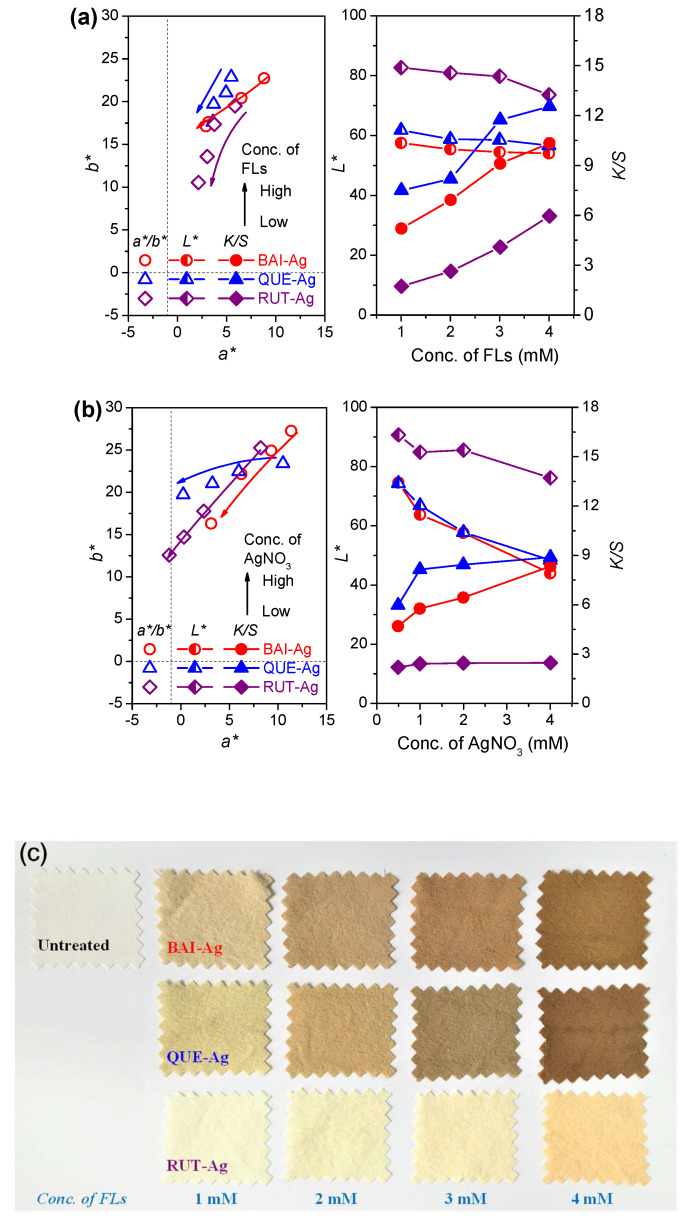
Color characterizations: (**a**) concentration of FLs, (**b**) concentration of AgNO_3_ and (**c**) photos.

Figure 7a–c illustrate the a*/b* coordinates for *QUE-Ag@Silk*, *BAI-Ag@Silk*, and *RUT-Ag@Silk* samples based on CIE colorimetric measurements. Untreated silk fabric appeared transparent with high reflectivity, positioning its a*/b* values close to the zero point of coordinate system. Upon the generation of AgNPs, the textile substrates developed a distinct brown coloration. Higher concentrations of flavonoid compounds and silver nitrate caused the a*/b* data points to migrate toward the graph’s center, indicating reduced chromatic intensity and muted visual characteristics. BAI-Ag displays a slightly stronger red hue and darker shade on silk than QUE-Ag and RUT-Ag (Figure 7a), which is due to the higher quantity of AgNPs generation on silk as well as the color of BAI itself. The photographic evidence in the Figure demonstrates how FLs-Ag@Silk samples undergo chromatic transitions with varying silver nitrate concentrations. Progressive deposition of metallic AgNPs on the textile matrix resulted in intensified pigmentation and darker hues, correlating directly with elevated concentrations of both silver nitrate and flavonoid solutions.

### 3.5. Functionality

#### 3.5.1. Bioactivity

**Figure 8:**
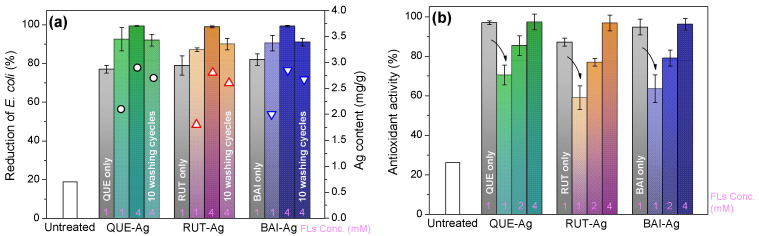
(**a**) Antimicrobial and (**b**) Antioxidant activities. (Note: circles and triangles represent the Ag content of samples; arrows stand for the decrease of antioxidant activity; pink numbers are FLs concentration used for sample preparation.).

The antimicrobial performance of FLs-Ag@Silk against *E. coli* was thoroughly examined. Figure 8a reveals that untreated silk fabric exhibited minimal antibacterial effects, achieving merely an 18% reduction in bacterial colonies. While FLs treatment enhanced silk’s antimicrobial properties, the effectiveness remained under 80%. In contrast, FLs-Ag@Silk demonstrated outstanding bactericidal performance. Specifically, silk modified through in situ silver nanoparticle deposition achieved over 92% inhibition of *E. coli* within 24 h. Similar trend of bacterial reduction also occurs against *S. aureus*. (Figure S4) This remarkable antibacterial capability originates from multiple mechanisms. Silver nanoparticles adhere to microbial cell walls, disrupting sulfur-rich membrane proteins. They further infiltrate bacterial cells, interfering with phosphorus-based biomolecules including genetic material. Additionally, silver ions gradually released from the nanoparticles contribute significantly to the antimicrobial effect. Beyond silver’s role, residual FLs absorbed during processing also participate in bacterial elimination. Remarkably, after undergoing ten washing cycles, FLs-Ag@Silk maintained over 95% of its initial antibacterial efficiency rising from the very small quantity of AgNPs release confirmed by ICP analysis, which is below the toxicity level [18]. This durability confirms the stable integration of silver nanoparticles achieved through the in situ synthesis approach.

Figure 8b demonstrates that raw silk exhibits minimal antioxidant performance, measuring only 25%. Following FLs application, the material’s radical inhibition capacity significantly improves, reaching 95–98%. Interestingly, FLs-Ag@Silk shows reduced antioxidant effectiveness initially, though this property enhances proportionally with higher FLs dosage. The underlying mechanism involves oxidation of catechol’s phenolic hydroxyl groups (located in flavonoids’ B ring) during silver nanoparticle formation, transforming them into quinone structures [15]. Furthermore, unbound hydroxyl groups within the FLs A-ring maintain residual radical-neutralizing capabilities, partially sustaining the modified silk’s antioxidative properties.

#### 3.5.2. UV Protection

**Figure 9:**
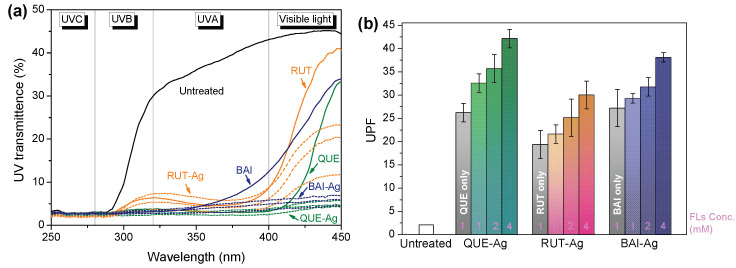
(**a**) UV transimittence and (**b**) UPF values of untreated and treated silk. (Note: pink numbers are FLs concentration used for sample preparation.).

The UPF measurement quantifies a material’s capacity to shield against solar ultraviolet rays, determined by comparing the average effective skin irradiance with that blocked by the textile. This rating derives from assessing light transmission percentages across both UVA (315–400 nm) and UVB (280–315 nm) wavelength bands. Textiles achieving UPF scores exceeding 15 demonstrate adequate shielding, while those surpassing 40 offer outstanding UV defense according to AS/NZS 4399 (1996) standards. Figure 9 presents the UV-blocking performance of FLs-Ag@Silk through UPF metrics. Untreated silk exhibits minimal protection, registering a mere UPF of 3. The incorporation of FLs elevates this protection to a range of 20–25. When FLs concentration increases, the treated fabrics attain superior UV resistance as silver nanoparticle (AgNP) formation intensifies. The exceptional UV shielding provided by FLs-Ag@Silk significantly mitigates photoaging effects and minimizes skin damage from solar ultraviolet exposure.

## 4. Conclusions

Making full use of agricultural byproducts for the dyeing and functionalization of textiles is greatly encouraged for moving towards carbon neutralizing and economizing targets. In this study, in situ generation and immobilization of AgNPs on silk through one-step bio-reduction by three representative FLs, i.e., BAI, QUE and RUT were developed. Results showed flavonoids-derived AgNPs@silk produced a beautiful color palette ranging from light creamish brown to dark golden brown. In the presence of equivalent dosage of flavonoids on silk, the color depth follows the order: QUE-Ag@silk > BAI-Ag@silk > RUR-Ag@silk due to the descending reactivity of these FLs and their fastness on silk fiber. Morphological images show that AgNPs are spherical in shape and well dispersed on silk. The AgNPs were highly monodispersed with an average particle size of 20–40 nm. The particle size of AgNPs decreased with increasing concentration of FLs and decreasing concentration of AgNO_3_. All the FLs-Ag@silk exhibit outstanding antibacterial activity (>98% bacterial reduction) and excellent laundering durability, where it inhibits >94% of *E. coli* even after 10 washing cycles. Moreover, AgNPs@silk is highly effective blocking of UV radiation in both UVA and UVB regions, thus offering excellent UV protection. The vitality of this research lies in the sustainable and eco-friendly synthesis of AgNPs using FLs, which is beneficial for the preparation of hygiene-related and medical textiles.

## Figures and Tables

**Table 1:** Matrix for variables and levels.

Variables	Levels				
	−α	−1	0	1	α
A: Conc-FLs (% owf)	1.32	2	3	4	4.68
B: Conc-Ag (mmol/L)	0.53	0.8	1.2	1.6	1.87
C: Temp. (°C)	53.18	60	70	80	86.82

**Table 2:** Experimental runs and responses.

Runs	Factors			*K/S*		
	Conc-FLs	Conc-Ag	Temp.	QUE	BAI	RUT
**1**	2	0.8	60	4.16	3.36	1.40
**2**	4	0.8	60	6.87	5.29	2.15
**3**	2	1.6	60	6.42	5.01	2.08
**4**	4	1.6	60	10.24	7.49	3.02
**5**	2	0.8	80	5.26	3.88	1.62
**6**	4	0.8	80	8.28	5.39	2.26
**7**	2	1.6	80	7.37	5.28	2.18
**8**	4	1.6	80	10.87	7.70	3.11
**9**	1.32	1.2	70	5.36	4.15	1.69
**10**	4.68	1.2	70	11.43	7.96	3.22
**11**	3	0.53	70	4.23	2.88	1.16
**12**	3	1.87	70	8.88	6.23	2.50
**13**	3	1.2	53.18	7.37	6.06	2.51
**14**	3	1.2	86.82	8.10	6.53	2.65
**15**	3	1.2	70	8.92	6.34	2.62
**16**	3	1.2	70	8.74	6.61	2.71
**17**	3	1.2	70	8.18	6.15	2.52
**18**	3	1.2	70	8.20	6.35	2.61
**19**	3	1.2	70	8.50	6.53	2.63
**20**	3	1.2	70	8.60	6.29	2.61

**Table 3:** Analysis of Variance for *QUE-Ag@Silk*.

Source	DF	Adj SS	Adj MS	F-Value	*p*-Value
Model	9	74.3126	8.2570	90.31	0.000
Linear	3	65.8738	21.9579	240.15	0.000
Conc-FLs	1	39.6783	39.6783	433.96	0.000
Conc-Ag	1	24.1165	24.1165	263.76	0.000
Temp.	1	2.0790	2.0790	22.74	0.001
Square	3	8.0169	2.6723	29.23	0.000
Conc-FLs×Conc-FLs	1	0.0518	0.0518	0.57	0.469
Conc-Ag×Conc-Ag	1	7.2757	7.2757	79.57	0.000
Temp×Temp	1	1.2465	1.2465	13.63	0.004
2-Way Interaction	3	0.4219	0.1406	1.54	0.265
Conc-FLs×Conc-Ag	1	0.3139	0.3139	3.43	0.094
Conc-FLs×Temp	1	0.0000	0.0000	0.00	0.986
Conc-Ag×Temp	1	0.1079	0.1079	1.18	0.303
Error	10	0.9143	0.0914		
Lack-of-Fit	5	0.4882	0.0976	1.15	0.442
Pure Error	5	0.4261	0.0852		
Total	19	75.2270			
**Model Summary**					
S	R-sq		R-sq (adj)	R-sq (pred)	
0.302380	98.78%		97.69%	94.15%	

**Table 4:** Analysis of Variance for *BAI-Ag@Silk*.

Source	DF	Adj SS	Adj MS	F-Value	*p*-Value
Model	9	35.9738	3.9971	142.99	0.000
Linear	3	28.9191	9.6397	344.85	0.000
Conc-FLs	1	15.9285	15.9285	569.83	0.000
Conc-Ag	1	12.7290	12.7290	455.37	0.000
Temp.	1	0.2616	0.2616	9.36	0.012
Square	3	6.7554	2.2518	80.56	0.000
Conc-FLs×Conc-FLs	1	0.3235	0.3235	11.57	0.007
Conc-Ag×Conc-Ag	1	6.6546	6.6546	238.06	0.000
Temp×Temp	1	0.0568	0.0568	2.03	0.185
2-Way Interaction	3	0.2993	0.0998	3.57	0.055
Conc-FLs×Conc-Ag	1	0.2681	0.2681	9.59	0.011
Conc-FLs×Temp	1	0.0286	0.0286	1.02	0.335
Conc-Ag×Temp	1	0.0025	0.0025	0.09	0.770
Error	10	0.2795	0.0280		
Lack-of-Fit	5	0.1390	0.0278	0.99	0.504
Pure Error	5	0.1405	0.0281		
Total	19	36.2533			
**Model Summary**					
S	R-sq		R-sq(adj)	R-sq(pred)	
0.167192	99.23%		98.54%	96.47%	

**Table 5:** Analysis of Variance for *RUT-Ag@Silk*.

Source	DF	Adj SS	Adj MS	F-Value	*p*-Value
Model	9	5.78333	0.64259	185.94	0.000
Linear	3	4.53025	1.51008	436.96	0.000
Conc-FLs	1	2.49858	2.49858	723.00	0.000
Conc-Ag	1	1.98923	1.98923	575.61	0.000
Temp.	1	0.04244	0.04244	12.28	0.006
Square	3	1.21923	0.40641	117.60	0.000
Conc-FLs×Conc-FLs	1	0.06344	0.06344	18.36	0.002
Conc-Ag×Conc-Ag	1	1.19691	1.19691	346.34	0.000
Temp×Temp	1	0.00671	0.00671	1.94	0.194
2-Way Interaction	3	0.03385	0.01128	3.27	0.068
Conc-FLs×Conc-Ag	1	0.02913	0.02913	8.43	0.016
Conc-FLs×Temp	1	0.00200	0.00200	0.58	0.464
Conc-Ag×Temp	1	0.00272	0.00272	0.79	0.396
Error	10	0.03456	0.00346		
Lack-of-Fit	5	0.01795	0.00359	1.08	0.467
Pure Error	5	0.01661	0.00332		
Total	19	5.81789			
**Model Summary**					
S	R-sq		R-sq(adj)	R-sq(pred)	
0.0587865	99.41%		98.87%	97.22%	

## Data Availability

The raw data supporting the conclusions of this article will be made available by the authors on request.

## Supplementary Materials

The following supporting information can be downloaded at: https://www.mdpi.com/article/10.3390/ma18235409/s1, Figure S1: Contour plots for the K/S values of RUT-Ag@Silk as functions of conc. of FLs, conc. of Ag+ and Temp.; Figure S2: TEM images for (a) QUE-Ag, (b) BAI-Ag and (c) RUT-Ag; Figure S3: Area shrinkage of QUE-Ag@silk, BAI-Ag@silk and RUT-Ag@silk; Figure S4: Reduction of S. aureus of QUE-Ag@silk, BAI-Ag@silk and RUT-Ag@silk.
