# Supplementary material for: Reutilizing Flavonoids from Agricultural By-Products for In Situ Generation and Immobilization of AgNPs on Silk Towards Coloration, Antimicrobial and Anti-UV Functions
Source: Materials (Basel). 2025 Nov 30;18(23):5409. doi: 10.3390/ma18235409 (PMC12693654; doi:10.3390/ma18235409)
Supplement: Supplementary file 1 [file materials-18-05409-s001.zip › materials-3972775-supplementary.pdf]

Supporting Information for

Reutilizing Flavonoids from Agricultural By-Products for In Situ Generation and Immobilization of AgNPs on Silk Towards Coloration, Antimicrobial and Anti-UV Functions

Wei Chen [1], Yijie Yue [1], Xiaoqi Zhou [1], Jingyu Sun [1], Leyang Chen [2], Xiaoyan Hu [1,*] and Yuyang Zhou [2,*]

[1] School of Art, Soochow University, Suzhou 215123, China; chenweisdyz@163.com (W.C.); yueyijie@suda.edu.cn (Y.Y.)
[2] College of Textile and Clothing Engineering, Soochow University, Suzhou 215123, China
* Correspondence: huxiaoyi0214@163.com (X.H.); yuyangzhou@suda.edu.cn (Y.Z.)

Four figures in page S2, S3

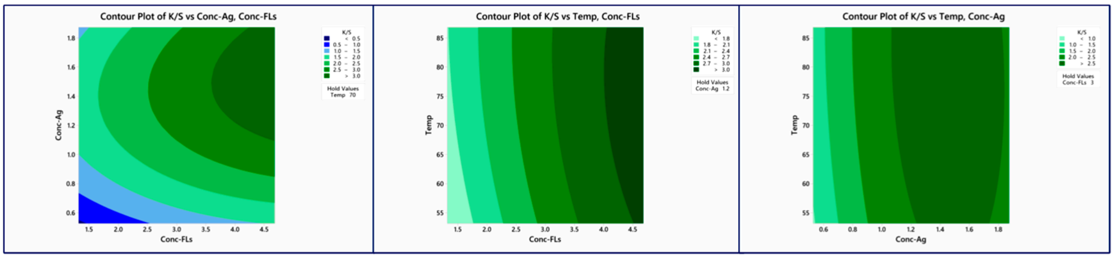

Figure S1 Contour plots for the *K/S* values of RUT-Ag@Silk as functions of conc. of FLs, conc. of Ag^{+} and Temp.

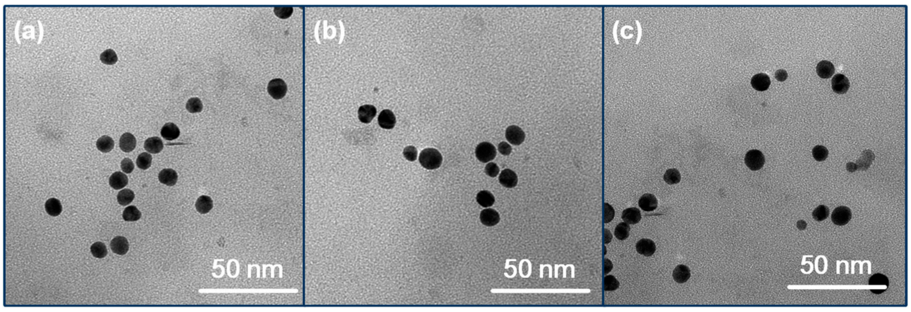

Figure S2 TEM images for (a) QUE-Ag, (b) BAI-Ag and (c) RUT-Ag.

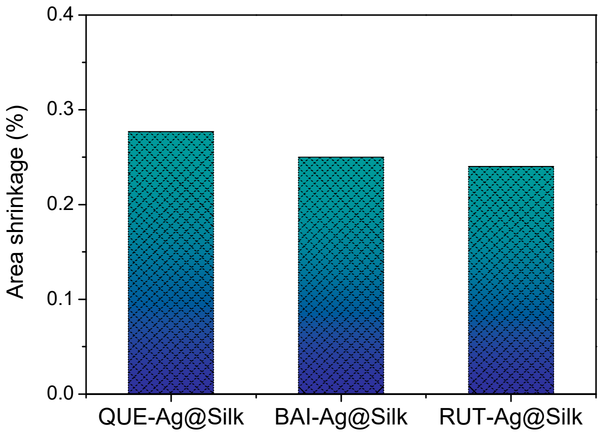

Figure S3 Area shrinkage of QUE-Ag@silk, BAI-Ag@silk and RUT-Ag@silk.

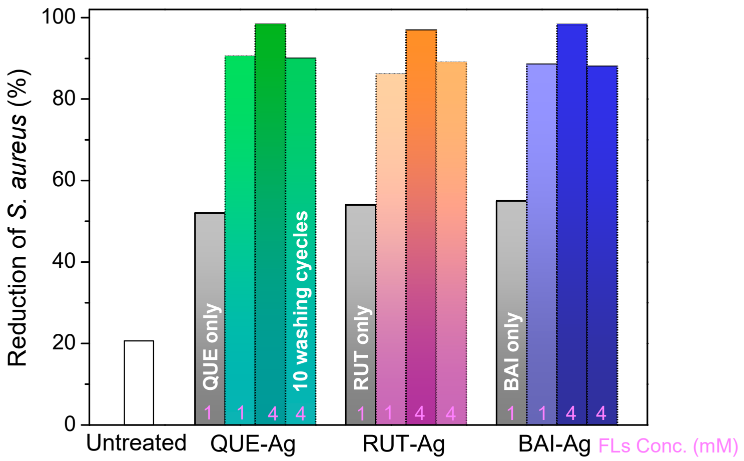

Figure S4 Reduction of *S. aureus* of QUE-Ag@silk, BAI-Ag@silk and RUT-Ag@silk.
